# Ir-LBP, an *Ixodes ricinus* Tick Salivary LTB4-Binding Lipocalin, Interferes with Host Neutrophil Function

**DOI:** 10.1371/journal.pone.0003987

**Published:** 2008-12-19

**Authors:** Jérôme Beaufays, Benoît Adam, Catherine Menten-Dedoyart, Laurence Fievez, Amélie Grosjean, Yves Decrem, Pierre-Paul Prévôt, Sébastien Santini, Robert Brasseur, Michel Brossard, Michel Vanhaeverbeek, Fabrice Bureau, Ernst Heinen, Laurence Lins, Luc Vanhamme, Edmond Godfroid

**Affiliations:** 1 Laboratory for Molecular Biology of Ectoparasites, IBMM, Université Libre de Bruxelles, Gosselies, Belgium; 2 Centre de Biophysique Moléculaire Numérique, Gembloux Agricultural University, Gembloux, Belgium; 3 Institute of Human Histology, Department of Morphology and Immunology, Faculty of Medicine, University of Liège, Liège, Belgium; 4 Laboratory of Cellular and Molecular Physiology, GIGA-Research, University of Liège, Liège, Belgium; 5 Laboratoire de Médecine Expérimentale (ULB 222 Unit), ISPPC Hopital André Vesale, Montigny-Le-Tilleul, Belgium; 6 Institute of Zoology, University of Neuchâtel, Neuchâtel, Switzerland; 7 Laboratory of Molecular Parasitology, IBMM, Université Libre de Bruxelles, Gosselies, Belgium; Centre de Recherche Public-Santé, Luxembourg

## Abstract

**Background:**

During their blood meal, ticks secrete a wide variety of proteins that can interfere with their host's defense mechanisms. Among these proteins, lipocalins play a major role in the modulation of the inflammatory response.

**Methodology/Principal Findings:**

We previously identified 14 new lipocalin genes in the tick *Ixodes ricinus*. One of them codes for a protein that specifically binds leukotriene B4 with a very high affinity (Kd: ±1 nM), similar to that of the neutrophil transmembrane receptor BLT1. By *in silico* approaches, we modeled the 3D structure of the protein and the binding of LTB4 into the ligand pocket. This protein, called Ir-LBP, inhibits neutrophil chemotaxis *in vitro* and delays LTB4-induced apoptosis. Ir-LBP also inhibits the host inflammatory response *in vivo* by decreasing the number and activation of neutrophils located at the tick bite site. Thus, Ir-LBP participates in the tick's ability to interfere with proper neutrophil function in inflammation.

**Conclusions/Significance:**

These elements suggest that Ir-LBP is a “scavenger” of LTB4, which, in combination with other factors, such as histamine-binding proteins or proteins inhibiting the classical or alternative complement pathways, permits the tick to properly manage its blood meal. Moreover, with regard to its properties, Ir-LBP could possibly be used as a therapeutic tool for illnesses associated with an increased LTB4 production.

## Introduction

Neutrophils are key players in the inflammatory response as they constitute the first line of defense after infection or injury. They are involved in phagocytosis and the degradation of micro-organisms in the phagolysosome by the production of ROS (Reactive Oxygen Species), antimicrobial peptides and proteases such as elastase [1]. Neutrophils may also destroy pathogens without phagocytosis by secreting antimicrobial factors into the extracellular medium. Moreover, neutrophils play an essential role in the control of the response to non-infectious inflammatory stimuli. This control is carried out by serine proteases secreted at the inflammation site, which are involved in the activation and inactivation of chemokines and cytokines, activation of membrane receptors and cleavage of adhesion proteins [2]. Furthermore, neutrophils are recruited and activated at the inflammation site by various factors including, in particular, leukotriene B4 (LTB4), formyl methionyl leucyl phenylalanine (fMLP) [3], interleukin-8 (IL-8) [4], [5], anaphylatoxin C5a [6], [7] and Platelet Activating Factor (PAF) [8], [9]. During their blood meal, ticks can modulate this pro-inflammatory neutrophil activation by means of factors secreted by their salivary glands. For example, the saliva of the hard tick *Ixodes scapularis* inhibits inflammatory neutrophil response [10]. In addition, an anti-IL8 activity preventing the interaction of IL-8 with its neutrophil receptors was described in the salivary glands of several tick species (*Dermacentor reticulatus, Amblyomma variegatum, Rhipicephalus appendiculatus, Haemaphysalis inermis, Ixodes ricinus*) [11]. Similarly, a family of inhibitors of the alternative complement pathway, specifically targeting properdin, a positive regulator of C3 convertase, was identified and characterized in the tick *I. ricinus* [12], [13]. This activity prevents the production of C5a, an important inflammatory mediator. Recently, Mans and Ribeiro have demonstrated that some salivary proteins (moubatin, TSGP2, TSGP3, TSGP4 and AM-33) from soft tick species (*Ornithodoros moubata*, *Argas monolakensis*) are able to scavenge arachidonic acid derivatives such as thromboxane A2 (moubatin and TSGP3), LTB4 (moubatin, TSGP2 and TSGP3) or leukotriene C4, D4 and E4 (TSGP4 and AM-33) with high affinity [14], [15].

Leukotriene B4 (LTB4) is a main actor in the recruitment and activation of neutrophils [16]. It allows hyperadhesiveness of neutrophils to endothelial cells [17], [18], and induces degranulation [19], superoxide anion secretion [20] and delayed apoptosis [21]. LTB4 is a small lipid molecule generated from arachidonic acid via the 5-lipoxygenase pathway. Cell activation by LTB4 involves 2 protein G receptors called BLT1 and BLT2. Receptor BLT1 is mainly expressed on the surface of leukocytes [22] whereas BLT2 expression is more ubiquitous, as it is observed on the surface of several cell types [23]. These two receptors have different affinities for LTB4. BLT1 has a dissociation constant (Kd) of about 1.0 nM [24]–[26] whereas BLT2 has a Kd of about 23 nM [23]. Lastly, LTB4 has many other functions in addition to its major role in neutrophil activation. For instance, it is involved in the recruitment and activation of eosinophils [27], T-lymphocytes [28]–[30], monocytes [31], mastocytes [32] and dendritic cells [33], thereby playing a central role during the development of the inflammatory response.

In the previous study, we described the identification of 14 sequences (Lipocalin from *Ixodes ricinus*; LIR1 to LIR14) belonging to the lipocalin superfamily in the tick *I. ricinus*. Convergent experimental and *in silico* approaches allowed us to define one of these proteins, LIR6, as an LTB4 binding protein. In accordance with the nomenclature used for the three histamine-binding proteins from the saliva of *R. appendiculatus* ticks (Ra-HBP), LIR6 was renamed Ir-LBP for “*Ixodes ricinus* leukotriene B4-binding protein”. We demonstrated that the protein Ir-LBP specifically binds to LTB4 with a similar affinity to that of BLT1 and can therefore act as an LTB4 scavenger. *In silico* analysis allowed to model its structure supporting its specificity for LTB4. Moreover, Ir-LBP interferes with neutrophil chemotaxis and apoptosis induced by LTB4. Lastly, *in vivo* experiments established the anti-inflammatory role of Ir-LBP through its action on neutrophils located at the tick bite site.

## Results

### Biochemical characterization of Ir-LBP

**Figure 1:**
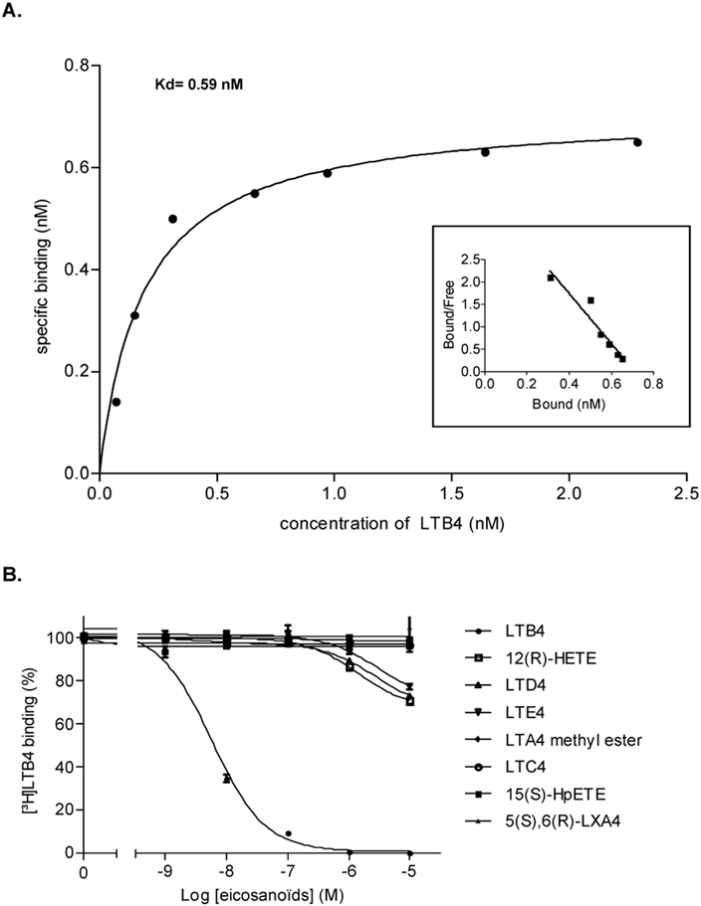
Binding of ^3^H-LTB4 to Ir-LBP. a) Saturation curve and scatchard analysis for LTB4 binding to Ir-LBP. b) Inhibition of ^3^H-LTB4 binding to Ir-LBP by eicosanoïds. The binding of 1 nM ^3^H-LTB4 was subjected to competition with the indicated concentrations of the indicated eicosanoïds.

We previously described the cloning and the expression of recombinant Ir-LBP (previously named LIR6), a salivary tick protein belonging to the lipocalin superfamily, which is able to bind specifically leukotriene B4. We therefore undertook a refined analysis of this binding. For this purpose, we produced a recombinant form of Ir-LBP expressed in insect Sf9 cells, and purified it by affinity chromatography on a Ni2+ chelate resin (see Materials and Methods). The GenBank (http://www.ncbi.nlm.nih.gov/Genbank) accession number for Ir-LBP protein is AM055950. Highly purified recombinant Ir-LBP was first used to determine the dissociation constant of Ir-LBP for LTB4 by incubating it with increasing concentrations of ^3^H-LTB4. The binding of LTB4 to Ir-LBP (three independent measurements) was saturable with a Kd value of 0.59 nM±0.57 (Figure 1A). This value is close to that obtained for one of the 2 membrane receptors specific to LTB4, namely BLT1, showing that Ir-LBP has a high affinity for LTB4. Moreover, the linearity of Scatchard plot analysis indicated the presence of a single high affinity LTB4 binding site on Ir-LBP.

The specificity of this binding was then evaluated by incubating Ir-LBP with increasing concentrations of different eicosanoids (LTB4, LTD4, LTC4, LTE4, 12(R)-hydroxyeicosatetraenoic acid (HETE), 15(S)-hydroxyperoxyeicosatetraenoic acid (HpETE), LTA4 methyl ester and 5 (S), 6 (R)-Lipoxin A4) in the presence of a fixed concentration of ^3^H-LTB4 (Figure 1B). The results show that only cold LTB4 is able to completely inhibit the binding of Ir-LBP to ^3^H-LTB4 (IC50 = 5.7 nM). On the other hand, only at a very high concentration of 10 µM were LTD4, LTE4 and 12(R)-HETE able to decrease the binding of 1 nM of ^3^H-LTB4 to Ir-LBP by approximately 30%. The other eicosanoids tested (LTA4 methyl ester, LTC4, 15(S)-HpETE and 5(S), 6(R)-Lipoxin A4) were unable to compete at this concentration. These results show that the scavenging of LTB4 by Ir-LBP is highly specific. Furthermore, this interaction is reversible. The addition of an excess of cold LTB4 (10 µM) for 2 h displaces the binding between Ir-LBP and 1 nM of ^3^H-LTB4 (data not shown). Overall, these results showed that Ir-LBP is a LTB4 scavenger protein binding to it with a very high affinity (±1 nM). This binding may only be displaced by very high concentrations (10 µM versus nM) of eicosanoid analogs.

### In silico modeling of Ir-LBP

#### Construction of 3D models–Analysis of the models

**Figure 2:**
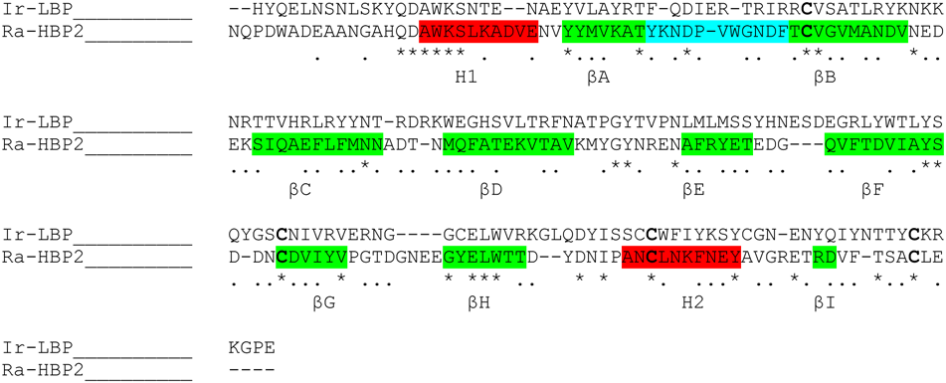
Alignment between Ir-LBP and Ra-HBP2 used as input for Modeler. * stands for an identical residue and • for similar residues. Secondary structure elements (βA to βI in green and H1, H2 in red; Ω loop in blue) of Ra-HBP2 are indicated.

In a previous study, we modeled LIR2 based on the refined alignment with Ra-HBP2 [34]. The same strategy was now used for Ir-LBP. The alignment presented in Figure 2 was used as input for the Modeler software. It should be noted that no gap was introduced in regular secondary elements. Four models were built for Ir-LBP. Their stereochemical validity was checked with Procheck [35]. No residue was found in the disallowed phi/psi regions of the Ramachandran plot (data not shown). In the models, the secondary structure is similar to that of the template; variations only occur in loops, as often for modeled structures. As for LIR2 [34], there is a potential disulfide bridge between Cys 187 and Cys 213, with no correspondence in Ra-HBP2.

**Figure 3:**
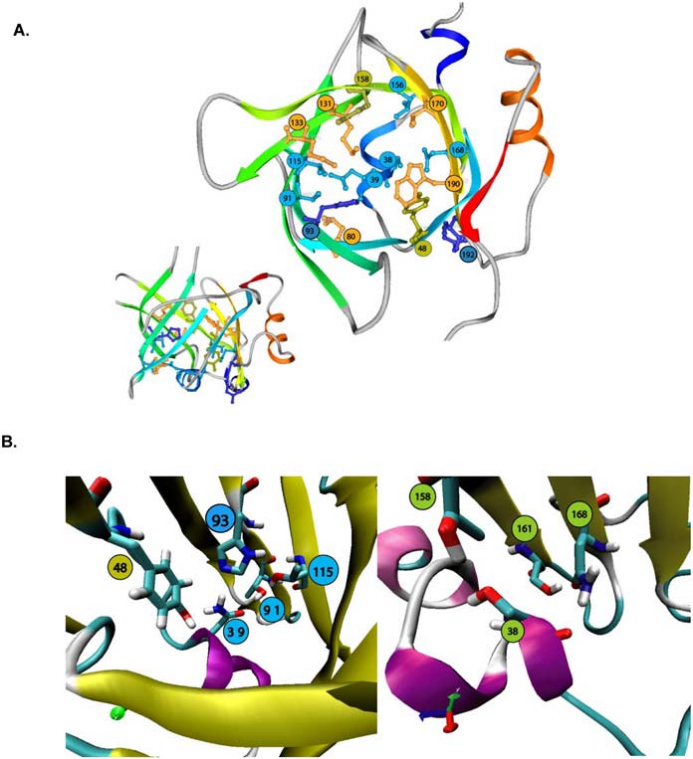
Model of Ir-LBP with the residues corresponding to the conserved internal lipocalin cluster. A. The protein is viewed from the top (right) and front (left). The protein is represented by a ribbon, colored by secondary structure succession. Positive residues are in blue, hydrophobic residues in orange, hydrophilic residues in light blue, and Tyr in green. The corresponding Ω loop is not represented for the sake of clarity. B. *Details of the bottom of the cavity of Ir-LBP*. The protein is represented by a ribbon. The strands are in yellow and H1 is in purple.

As mentioned, the internal cluster of conserved residues is more hydrophilic in Ir-LBP than the other LIRs or unrelated lipocalins (see the accompanying paper): residues 91 (Thr), 93 (His), 115 (Thr), 158 (Thr) and 168 (Asn) are hydrophilic, like the two conserved H1 residues (38 and 39). As shown in Figure 3B, residues Asn 39, Tyr 48, Thr 91, His 93 and Thr 115 could interact together and are thought to play a role in stabilizing the fold, similar to the hydrophobic residues at the same positions in other lipocalins.

It should be noted that Ir-LBP is significantly richer in positive residues than the other LIRs. Notably, the Arg content of Ir-LBP (10%) is largely superior to that of proteins in databanks (around 5%). For Ir-LBP, the corresponding Ω-loop, strand βC and the surrounding loops, as well as loop βE-βF, are particularly rich in charged residues. The mouth of the barrel contains more positive than negative residues as shown in Figure S2, and may therefore attract negatively charged ligands.

#### Analysis of the cavity

Globally, a segregation of residues liable to interact with a ligand is observed in the cavity of all LIRs; this is shown in Figure S1 for LIR2 and Ir-LBP. The top of the cavity is enriched in hydrophilic residues and the bottom is more hydrophobic. The profile of the cavity of LIRs suggests that they may bind hydrophobic ligands such as fatty acids and hence leukotrienes that are linear and have a hydrophobic tail. Three major leukotrienes are involved in homeostasis and inflammation: leukotrienes B4 (LTB4), C4 (LTC4) and D4 (LTD4). The latter two have a peptidic moiety bonded near the carboxyl end and one hydroxyl group on the aliphatic chain. For LTC4, the peptidic moiety is composed of Glu-Cys-Gly and Cys-Gly for LTD4. LTB4 has two hydroxyl groups on the aliphatic chain and no peptidic moiety. This could explain the specificity of Ir-LBP to LTB4.

#### Molecular dynamics simulation of the interaction of Ir-LBP with LTB4

**Figure 4:**
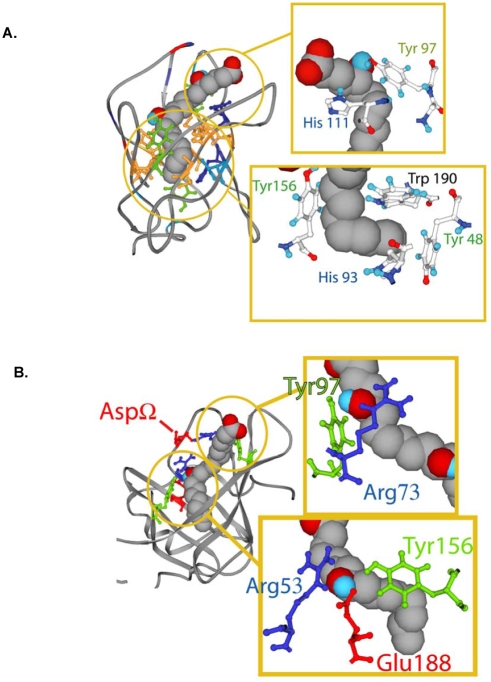
Snapshots of the molecular dynamics simulation of Ir-LBP interacting with LTB4. Ir-LBP is represented by a ribbon and LTB4 in CPK. Tyr are green and positive, negative and hydrophobic residues are blue, red and orange, respectively. A. Zoom on the carboxyl group and the hydrophobic tail of LTB4. B. Zoom on two hydroxyl groups of LTB4.

To obtain some insight into the molecular interactions between Ir-LBP and LTB4, molecular dynamics simulations were carried out using Gromacs [36]. Before the simulation, the LTB4-Ir-LBP complex was solvated and minimized. The dynamic simulations lasted 3ns, including an equilibration phase of about 1 ns. During simulations, the hydroxyl groups and hydrophobic moiety potentially interact with Ir-LBP cavity (figure 4A); all the Ir-LBP residues that are suggested by the model to interact with LTB4 are represented in Figure 3. On the other hand, the carboxyl group of LTB4 is situated at the mouth of the barrel and is predicted to interact with the solvent and the loops (figure 4B). Calculations suggest that one hydroxyl group of LTB4 interacts with Arg 73 and Tyr 97 and the second one interacts with Arg 53, Tyr 156 and Glu 188 (Figure 4B). The aliphatic tail is predicted to interact with hydrophobic residues at the bottom of the barrel and two histidines. As shown in Figure 4A, they are mainly aromatic and might play an important role in stabilizing the hydrophobic moiety.

The other LIRs do not bind LTB4 “*in vitro*”, meaning that the residues involved in LTB4 binding are not well conserved. Residue 73 is not conserved as positive; 97 is not conserved as hydrophilic, except for LIR2 and LIR11; 53 is conserved as positive only for LIR1, 7 and 11; the nature of residues 156 and 188 (hydrophilic and negative, respectively) is conserved for all LIRs. The residues suggested by the modeling to be involved in the stabilization of the hydrophobic part of LTB4 are better conserved as they are aromatic for all LIRs, except for residue 156. This conservation analysis suggests that Arg 73 could play a role in LTB4 binding. Together with the fact that Ir-LBP has a high net positive charge, the presence of this residue may explain at least partially why Ir-LBP is the only LIR to bind LTB4.

In summary, our “*in silico*” analysis casts light on properties fully compatible with the experimental LTB4 binding: a barrel shaped structure, the composition of the cavity and the adequate location of critical amino-acid residues. It also suggests an explanation as to why Ir-LBP is able to bind LTB4, as opposed to other LIRs.

### Binding properties of selected mutants

In order to determine which amino acids of Ir-LBP are involved in the binding to LTB4, we produced mutants designed on the basis of the dynamic model established *in silico*. This model suggests that the interaction with the hydroxyl group of LTB4 takes place on the one hand via Arg 73 and Tyr 97, and on the other via Arg 53, Tyr 156 and Glu 188. Arg 73 was chosen to be mutated rather than Tyr 97 because it is not conserved in the other LIRs and should hence be specific for LTB4 binding. For the interaction with the second hydroxyl group, the residue selected to be mutated is Arg 73. The choice was made on this residue because Glu 188 is conserved for all LIRs and should thus not be as specific as Arg 73; furthermore, Arg 53 is bridged to Arg 73 by an aspartic residue from the loop corresponding to the Ω loop in Ra-HBP2. The two hydroxyl groups and the two Arg bridged by Asp Ω could form an interacting network, further stabilizing the binding with LTB4. Two single mutants were therefore produced by replacing Arg 53 with threonine (R53T) and Arg 73 with alanine (R73A). A double mutant (R53T/R73A) was also produced by combining the 2 substitutions of the single mutants. A null mutant was also constructed by replacing serine 124 with threonine (S124T). Serine 124 is located close to the interaction pocket but does not seem to be involved in binding with LTB4. Therefore, the affinity of this mutant for LTB4 should not be affected. These mutants and “wild type” Ir-LBP were expressed in 293T cells, and analyzed to determine their dissociation constant for LTB4. As expected, the null mutant S124T had an affinity (Kd = 0.45 nM±0.01) for LTB4 similar to the “wild type” Ir-LBP (Kd = 0.52 nM±0.11). Mutant R53T showed no loss of affinity for LTB4 (Kd = 0.43 nM±0.07) whereas that of mutant R73A increased approximately 1.7 times (Kd = 0.87 nM±0.13). The dissociation constant of the double mutant was increased by approximately 10 times (Kd = 4.23 nM±0.27). These results suggest that Arg at position 73 is involved in LTB4 binding, while the involvement of Arg at position 53 seems enhanced in the frame of the double mutant configuration (R53T/R73A).

### In vitro activity of Ir-LBP

#### Ir-LBP inhibits delayed neutrophil apoptosis induced by LTB4

**Figure 5:**
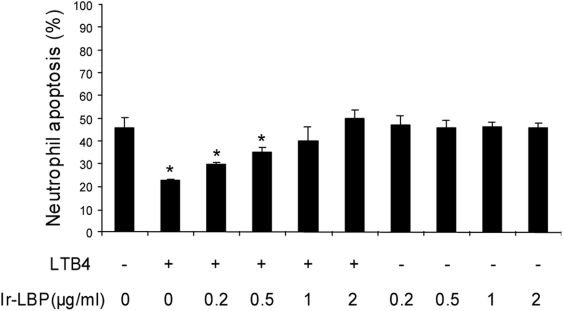
Ir-LBP inhibits the anti-apoptotic effects of LTB_4_ in neutrophils. Human neutrophils were cultured for 24 h in the absence or presence of LTB_4_ (10^−7^ M), graded concentrations of Ir-LBP (0.2, 0.5, 1 and 2 µg/ml), or both LTB_4_ (10^−7^ M) and Ir-LBP (0.2 to 2 µg/ml). Apoptosis was assessed by Annexin-V-FITC/PI staining and flow cytometry analyses. * Significantly different from the results obtained with untreated cells (*p*<0.05).

Neutrophils have a very short half-life (8–20 h) as they undergo constitutive apoptosis, a process ensuring the elimination of dead cells without the release of cytotoxic granules into the surrounding tissues [37]. The inflammatory response is accompanied by an expansion of the peripheral neutrophil pool as a result of enhanced granulopoiesis and decreased neutrophil apoptosis [38]. Among the different inflammatory mediators, LTB4 is well known for its ability to delay neutrophil apoptosis [21]. We therefore asked if Ir-LBP could counteract the anti-apoptotic effects of LTB4. For that purpose, enriched neutrophil populations were co-incubated in culture with LTB4 and Ir-LBP. After 24 h of culture, apoptosis levels were significantly lower in LTB4-treated neutrophils than untreated controls (Figure 5). Addition of increasing concentrations of Ir-LBP to the culture medium gradually inhibited LTB4-induced neutrophil survival (Figure 5). The anti-apoptotic effects of LTB4 were completely abolished when 1 and 2 µg/ml of Ir-LBP were used (Figure 5). Ir-LBP alone, even at higher concentrations, had no measurable effects on neutrophil apoptosis, even when higher Ir-LBP concentrations were used (Figure 5). Taken together, these results show that Ir-LBP is capable of inhibiting the anti-apoptotic effects of LTB4.

#### Ir-LBP inhibits neutrophil transendothelial migration induced by LTB4

**Figure 6:**
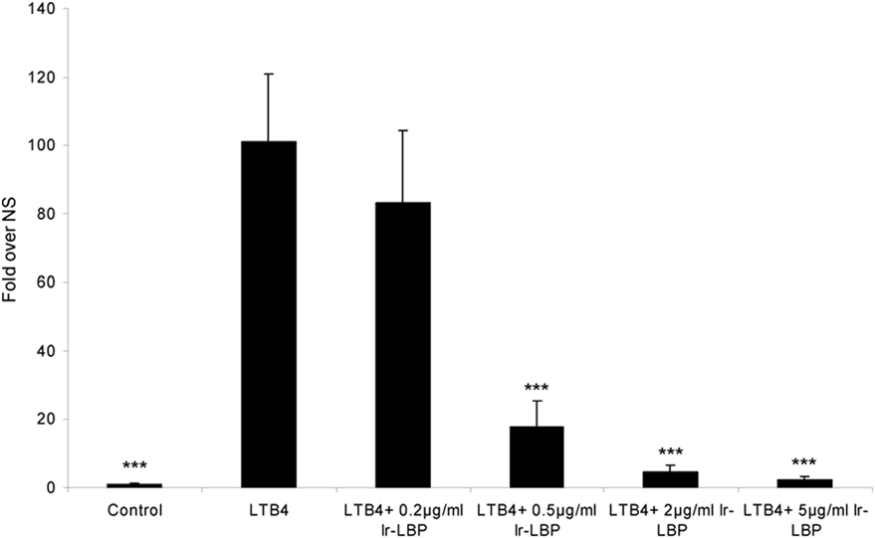
Ir-LBP effect on transendothelial migration of freshly isolated neutrophils stimulated by LTB4. Results represent the number of cells that cross the endothelial barrier after 2 h. Results were obtained from two independent experiments and are expressed as a fold increase over control. One LTB4 concentration and four Ir-LBP concentrations, 10E^−7^ M and a range from 0.2 µg/ml to 5 µg/ml respectively were used.

One of the major actions of LTB4 is chemotaxis of neutrophils to the inflammation site [16]. We therefore sought to measure the effect of Ir-LBP on the diapedesis aspect of this process by using a model of transendothelial migration described in 1997 by Nohgawa and co-workers [39]. Figure 6 shows the results of two separate experiments. White blood cells were obtained twice from a single volunteer at a 6 week interval. Ir-LBP was indeed shown to have a dose-dependent effect (starting at 0.5 µg/ml) on the transendothelial migration of neutrophils. Neutrophil mortality determined by trypan blue after 2 h of incubation was less than 5%, under each condition (data not shown).

### In vivo activity of Ir-LBP

**Figure 7:**
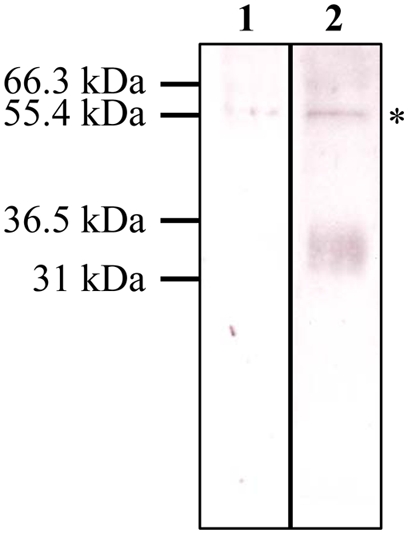
Western blot analysis of sera from repeatedly bitten rabbits. Five hundred nanograms of recombinant Ir-LBP were submitted to SDS-PAGE and analyzed by western blot using pooled serum from two naïve rabbits (lane 1) and rabbits infested three times by *I. ricinus* female ticks (lane 2). *: unspecific band.

**Figure 8:**
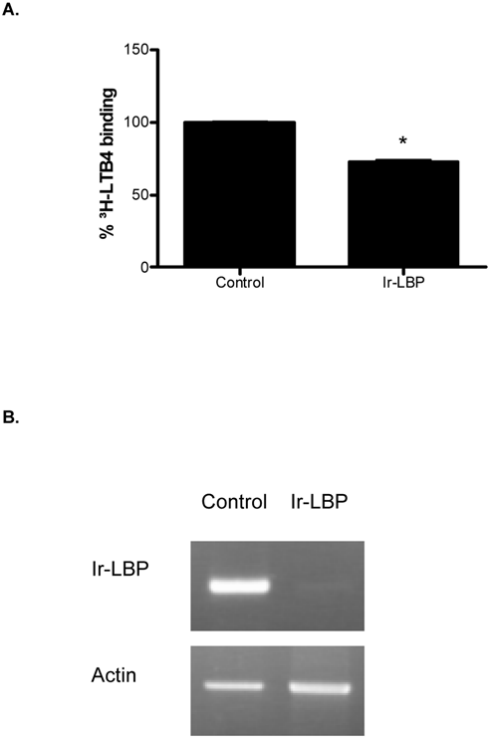
Ir-LBP knockdown in tick salivary glands. A) Eight pairs of salivary glands were incubated for 6 h with control or Ir-LBP siRNA. Five µg of salivary gland extracts were incubated with 2 nM of ^3^H-LTB4. B) RT-PCR analysis of RNA from control and Ir-LBP siRNA-treated salivary glands using Ir-LBP or actin gene-specific primers.

In order to evaluate the *in vivo* role of Ir-LBP, we first assessed its secretion by tick salivary glands. For that purpose, purified recombinant Ir-LBP was analyzed using sera from rabbits repeatedly infested by female *I. ricinus* ticks. Figure 7 shows that recombinant Ir-LBP protein was effectively recognized by these sera indirectly, showing that natural Ir-LBP protein is secreted in the saliva during infestation. We then carried out RNA inhibition experiments *in vivo* in the tick. Thus, we evaluated the effect of siRNA injection in the tick, both on the capacity of salivary gland extracts to bind LTB4 and on the density, morphology and survival of neutrophils at the bite site. First, siRNAs specifically targeting *Ir-LBP* mRNA were synthesized and their effectiveness in inhibiting the expression of recombinant protein Ir-LBP was demonstrated in the 293T mammalian cell system by cotransfecting siRNA and the plasmid Ir-LBP/pCDNA3.1/V5-His-TOPO (data not shown). Then, salivary glands isolated from 5-day engorged female ticks were incubated for 6 h with Ir-LBPsiRNA. RT-PCR analysis of mRNA isolated from these salivary glands showed a strong reduction in *Ir-LBP* mRNA level compared to the actin mRNA used as control (Figure 8B), confirming the results in the endogenous system and demonstrating its specificity. Five µg of protein extracts derived from these salivary glands were incubated with 2 nM of ^3^H-LTB4. The results showed a reduction of 28.25% (SD = 2.01) in the binding capacity of the extract of Ir-LBPsiRNA-treated glands compared to an extract from glands treated with a non-relevant control siRNA (Figure 8A).

**Figure 9:**
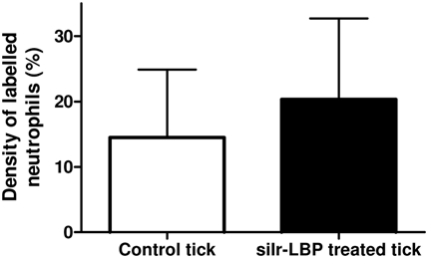
Density measurement of labeled neutrophils in skin of tick-infested mice. The light histogram represents the percentage of neutrophils per zone in skin sections of mice infested with control ticks (n = 55). The dark histogram represents the percentage of neutrophils per zone in skin sections of mice infested with ticks treated with Ir-LBP siRNA (n = 35, **: p<0.05).

**Figure 10:**
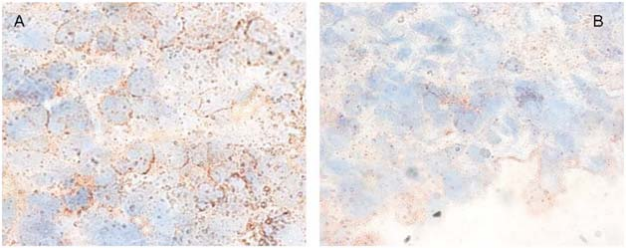
Nuclear morphology of immunolabeled neutrophils in the skin, close to the aspiration cavity. Panel A shows the enlarged nuclear morphology of neutrophils and intense immunolabeling using anti-mouse NIMP-R14 neutrophil antibody (in red) in a skin section of a mouse infested with a Ir-LBP siRNA-treated tick. Panel B illustrates the nuclear morphology and labeling aspect of neutrophils (in red) in a skin section of a mouse infested with a control tick (100×).

**Figure 11:**
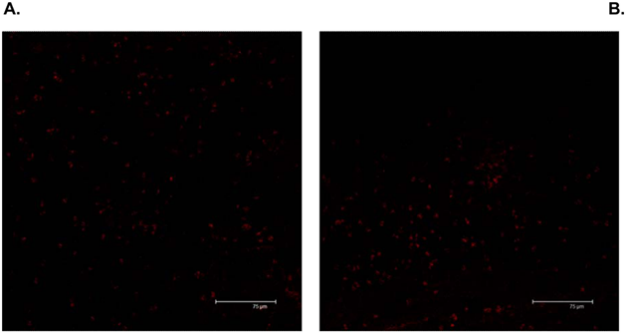
Fluorescence Detection of apoptotic cells. Panel A shows apoptotic cells (stained in red with the ApopTag kit) in a skin section of a mouse infested with a Ir-LBP siRNA-treated tick. Panel B shows apoptotic cells stained with the same technique in a skin section from a mouse infested with a siControlRNA-treated tick.

We also compared the density, morphology and survival of neutrophils in the skin of tick-infested mice with or without injecting silencing Ir-LBPsiRNA into the tick salivary gland. At the tick bite site, the rostrum was deeply implanted, rupturing the epidermis, dermis and hypodermis. Frequently, large accumulations of inflammatory cells, mainly neutrophils, were detected in the distended conjunctive tissue, especially around the blood aspiration cavity. We observed a larger number of neutrophils in the skin of mice infested with ticks treated by Ir-LBPsiRNA than in mouse skin infested with either untreated ticks or ticks treated with irrelevant siRNA or PBS. The neutrophil density, measured morphometrically after immunolabeling neutrophils, was significantly higher in skin infested with a tick treated with Ir-LBPsiRNA (p<0.05) (Figure 9). Enlarged neutrophils with dilated nuclei were found more often in the skin of mice infested with Ir-LBPsiRNA-treated ticks than under the other conditions. These neutrophils also appeared more intensely immunolabeled. This nuclear morphology and the increase of staining are well documented as being signs of cell activation (Figure 10). We did not observe any difference in the quantity of apoptotic cells counted under the different conditions. These apoptotic cells were mainly located in the hypodermis at a distance from the blood aspiration cavity (Figure 11).

## Discussion

We previously described a family of sequences coding for lipocalins in the salivary transcriptome of the hard tick *I. ricinus*. These proteins were called LIR for “Lipocalin from *I. ricinus*” and numbered from 1 to 14 (LIR1 to LIR14). Analysis of the transcriptome of different tick species suggested that lipocalins account for a large subgroup of the proteins expressed in the salivary glands. Numerous lipocalins have also been identified in both hard and soft ticks (*R. appendiculatus, D. reticulatus, I. scapularis*, *O. moubata*, *A. monolakensis*). Some of these (Ra-HBP, SHBP, IS-14 and IS-15) bind either histamine (Ra-HBP), 5-hydroxytryptamine (IS-14 and IS-15) or both histamine and 5-hydroxytryptamine (SHBP) [40]–[42]. Others scavenge thromboxane A2 (moubatin and TSGP3), LTB4 (moubatin, TSGP2 and TSGP3) or leukotriene C4, D4 and E4 (TSGP4 and AM-33) with high affinity [14]–[15]. In the accompanying paper, we attempted to determine the role of LIRs by measuring the binding capacity of these proteins to different ligands (histamine, 5-hydroxytryptamine, ADP norepinephrine, PAF, prostaglandins D2 and E2, and LTB4 and LTC4), known to act in the inflammatory response or, more generally, in hemostasis. Only LIR6 could specifically bind one of them, namely LTB4, and was therefore renamed Ir-LBP; the other LIRs bind neither LTB4 nor the other ligands tested. On the basis of *in silico* models constructed for the LIRs [34], we performed an in-depth analysis which provided clues about the structure, explaining the interaction between Ir-LBP and LTB4. Among all LIRs, Ir-LBP is the richest in positive residues and should therefore be more potent in attracting a negative ligand. By using dynamic simulations, we suggest that the two hydroxyl residues of LTB4 are interacting stably with 5 hydrophilic residues (2 Arg, 2 Tyr and 1 Glu), inside the barrel of Ir-LBP. Moreover, according to this model, the aliphatic tail of LTB4 is stabilized by hydrophobic interactions at the bottom of the barrel, and notably 4 aromatic residues (two Tyr, a His and a Trp). It should be noted that Arg 73, which interacts with the first hydroxyl group, is not conserved in any other LIR. Hence it is involved in the binding specificity of Ir-LBP and plays a key role in ligand binding as confirmed by mutational analysis (which also showed that it exerts its role in cooperation with Arg 53).

Two membrane receptors for LTB4, BLT1 and BLT2, have been identified so far. Their dissociation constant (Kd) values are approximately 1 nM for BLT1 and 23 nM for BLT2. In the present paper, we show that the binding of Ir-LBP to LTB4 is highly specific, reversible and saturable, and also that the high affinity (in the nM range) of Ir-LBP for LTB4 permits Ir-LBP to compete with LTB4 membrane receptors, thus impeding the transmission of the secondary messengers that are the initial steps of the cellular response.

In the course of an inflammatory response, LTB4 is one of the principal mediators involved in the recruitment and activation of neutrophils. It is responsible for neutrophil chemotaxis, adhesion to the blood vessel endothelium, transmigration, and degranulation, and promotes a delay in apoptosis. In this study, we showed that Ir-LBP influences endothelial transmigration of neutrophils, and delays their entrance into apoptosis induced by LTB4. During transmigration, LTB4 acts both on neutrophils, by inducing their migration, and on endothelial cells, by permitting diapedesis. LTB4 indeed induces the expression of CD54 protein (ICAM-1), allowing neutrophils to adhere to endothelial cells [17]. By sequestering LTB4, Ir-LBP prevents LTB4-dependent transmigration of neutrophils, and probably prevents the concomitant expression of proteins induced by LTB4, both in neutrophils and endothelial cells. Moreover, neutrophils have a half-life of 8 to 20 h and then constitutively enter apoptosis. However, apoptosis may be delayed by different mediators, such as LTB4. As expected, Ir-LBP inhibits the delay of neutrophil apoptosis induced by LTB4. Both Ir-LBP effects on transmigration and apoptosis are dose-dependent. At higher concentrations of Ir-LBP, the results are identical to those observed in the absence of LTB4, suggesting that Ir-LBP had captured all the available active LTB4. As a result, we infer that Ir-LBP, by binding LTB4, is a competitor for the host BLT1 and BLT2 membrane receptors. Similar results were observed with synthesized BLT1 and BLT2 antagonists. For example, ONO-4057, a BLT1 and BLT2 antagonist, totally inhibits endothelial transmigration of neutrophils [39]. Similarly, CP-105696 and SB201146, both BLT1 antagonists, are capable of inhibiting the apoptosis delay induced by LTB4 [43], [44].

Finally, we have also evaluated the role of Ir-LBP in the interaction of *I. ricinus* female ticks with their host. The recognition of recombinant Ir-LBP by sera from repeatedly bitten animals shows that their immune system has been in contact with the natural protein. This suggests that the protein is injected in the animal within the saliva. Moreover, we have also shown that *I. ricinus* salivary gland extracts harbour binding activity to LTB4. RNA interference experiments demonstrated the involvement of Ir-LBP in this activity as tick salivary glands submitted to *ex vivo* Ir-LBP siRNAs partially lose their ability to bind LTB4. Taken together, these results show that Ir-LBP acts *in vivo* as a LTB4 “scavenger”. Nevertheless, although the expression of Ir-LBP is inhibited, 70% of the LTB4-binding capacity remain. This suggests that there are probably other LTB4-binding factors. As mentioned in the accompanying paper, ticks express paralogue families that can fulfill the same function. It is therefore likely that there are other lipocalins (on top of non lipocalin proteins) able to bind LTB4 in the tick salivary gland.

*In vivo* RNAi was also performed by injecting Ir-LBP siRNA or irrelevant siRNA into ticks before putting them on mice. When skin biopsies were analyzed on cryosections five days later, clear differences were apparent between Ir-LBPsiRNA and control samples. Bite sites of Ir-LBPsiRNA-treated ticks appeared larger and more populated by neutrophils than those of control or irrelevant siRNA-treated ticks. We propose that the saliva of Ir-LBPsiRNA-treated ticks exerts a higher chemoattractant effect, likely as a consequence of a lower LTB4 scavenging potential. The neutrophils also appeared larger than those in control tick bite sites; in particular, the morphology of the nuclei was changed, appearing enlarged with less condensed chromatin. Here, too, it is likely that Ir-LBPsiRNA treatment, and therefore lower levels of Ir-LBP protein reduced the neutralization of LTB4, thus allowing a higher degree of activation of neutrophils. Ir-LBPsiRNA injection of the ticks also gave rise to more intense immunostaining of neutrophils around the bite site; since the antibody target in this assay is a membrane antigen, it is likely that yet another consequence of Ir-LBPsiRNA–treatment of ticks is a more efficient degranulation following activation of neutrophils. On the opposite, we could not determine any difference in the apoptotic status of neutrophils between control and Ir-LBPsiRNA conditions. This suggests that Ir-LBP does not influence neutrophil apoptosis *in vivo,* or alternatively that, *in vivo*, a reduction of apoptosis after Ir-LBPsiRNA treatment is hard to quantify due to the very low basal level of apoptotic neutrophils detected in normal situations. This low level might itself result from a continuous aspiration of blood and tissues removing living and dying cells. It has also been reported that activated neutrophils undergo a peculiar cell death process different from apoptosis [45] which could escape detection by the kit used. Finally, other molecules (than LTB4) such as TNFα or IL8 are released following tissue alteration by the tick rostrum, which can also modulate neutrophil apoptosis. They could on their own be sufficient, so that a change in LTB4 concentration would not influence neutrophil apoptosis.

According to our *in vivo* observations, tick saliva contains a factor able to reduce neutrophil chemotaxis and activation, which matches the *in vitro* ability of Ir-LBP to block LTB4. After Ir-LBP siRNA injection, however, the engorgement of the ticks was not affected in any means. Thus, *in vivo*, Ir-LBP delivered within the saliva could repress neutrophil influx and activation at the tick bite site, but this impairment appeared insufficient to alter the tick blood meal.

### Concluding remarks

This study addressed the biochemical function and physiological role in the tick blood meal of Ir-LBP. Ir-LBP has the capacity for very high affinity binding of LTB4, one of the principal mediators of the neutrophil-associated inflammatory response. *In vitro,* Ir-LBP competes with the natural LTB4 receptors in order to inhibit the transendothelial migration of neutrophils, as well as the apoptosis delay naturally induced by LTB4. *In vivo*, Ir-LBP is partially responsible for the LTB4 binding properties of tick salivary glands, as well as the reduction of neutrophil activation at the bite site. These elements show that Ir-LBP is a “scavenger” of LTB4, which, in combination with other factors, such as “histamine-binding protein” or proteins inhibiting the classical or alternative complement pathway, permits the tick to properly manage its blood meal. Moreover, with regard to its properties, Ir-LBP could possibly be used as a therapeutic tool for illnesses associated with an increase of the production of LTB4, such as Chronic Obstructive Pulmonary Disease (COPD), rheumatoid arthritis, psoriasis and inflammatory bowel disease, Crohn's disease and ulcerative colitis. Currently, BLT1 antagonists, such as LY29311, are being developed, but they may generate invalidating side effects due to their direct action on membrane receptors directly connected with the cellular machinery. Another therapeutic approach is to use LTB4 scavengers, like Ir-LBP. Later experiments will seek to determine the potential of Ir-LBP to become an effective therapeutic tool for any of these pathologies.

## Materials and Methods

### Ticks, salivary gland extracts and saliva

*Ixodes ricinus* ticks were bred and maintained at the University of Neuchâtel Institute of Biology (Switzerland). Colony founders were initially collected in the woodland near Neuchâtel and have been maintained on rabbits or Swiss mice for over 20 years. For the experiments described in this paper, pairs of adult (one female and one male) ticks were allowed to anchor and feed on rabbits for the indicated periods.

### Animals

Animal care and experimental procedures were carried out in accordance with the Helsinki Declaration (Publication 85-23, revised 1985), local institutional guidelines (laboratory license n° LA 1500474) and the Belgian law of August 14^th^, 1986 as well as the royal decree of November 14^th^, 1993 on the protection of laboratory animals. Studies were carried out using female New Zealand White Rabbits weighing about 3 kg obtained from Harlan (The Netherlands) and 8 week-old NMRI female mice weighing 20 to 25 g, obtained from Elevage Janvier (Le Genest-St-Isle, France).

### *In silico* approaches

#### Modeling of the LIRs 3D structure

3D models of LIRs were built using the Modeler program [46]. This method uses sequence homology between the protein of interest and a protein whose 3D structure is known to predict a three-dimensional model. The protein we use as model is Ra-HBP2 (pdb code: 1QFT). The primary alignment was obtained using ClustalW [47] and corrected by taking the conserved interactions of the lipocalin family (as described in [34]) into account. The resulting alignment is used as input for Modeler4.

The stereochemical quality of the 3D models is then checked using Procheck [35]). It contains 95% of ΦΨ angle pairs in the allowed regions of the Ramachandran plot, indicating a correct stereochemistry. The PROF prediction of secondary structure was obtained through the PredictProtein server (http://cubic.bioc.columbia.edu/predictprotein/) [48].

#### Molecular dynamics simulations

The molecular dynamics simulations were performed with the Gromacs 3.3 program [49], [36] using the Gromos 96 forcefield (43a2; with improved alkane dihedrals), for 3 ns at neutral pH. The LTB4 molecule was constructed in Hyperchem (release 7 for Windows-Hypercube Inc.). The charge distribution was calculated using a semi-empirical method. The structure was optimized with a steepest descent completed by the Polak-Ribiere conjugated gradient procedure. The LTB4 was then converted to the Gromacs format using PRODRG server and manually optimized to fit the forcefield [50].

The model was solvated in a cubic box (75/60/60 Å) with 7915 simple point charge (SPC) water molecules and simulated using periodic boundary conditions. Van der Waals interactions were truncated at 12 Å. The electrostatic interactions were treated using the particle mesh Ewald (PME) algorithm with a 10-Å truncation. The model was minimized by 2000 steps of steepest descent and 1000 steps of conjugate gradient and then equilibrated at the desired temperature and pressure for 50 ps, with all atom restraints except hydrogens, followed by 1 ns free of any atomic restraints. The time step for dynamics was 2.0 fs. The LINCS algorithm was used to satisfy bond constraints [51]. Temperature was controlled using a weak coupling to a bath of constant T (300K; coupling time of 0.1 ps) and pressure by a weak coupling to a bath of constant P (1 atm, coupling time of 0.5 ps) with the Berendsen method [36].

### Site-directed mutagenesis of Ir-LBP in pCDNA3.1/V5-His-TOPO

Mutants were produced by using the QuickChange PCR mutagenesis kit (Stratagene). The following PCR primers were used to generate R57T, R68A, S124T single mutants and the R57T-R68A double mutant: 5′-CAGAGTACGTACTGGCGTACACCACTTTTCAAGATATTGAAAG-3′ (R57Tforward), 5′-CTTTCAATATCTTGAAAAGTGGTGTACGCCAGTACGTACTCTG-3′ (R57Treverse), 5′-GATATTGAAAGGACACGTATTGCTAGATGCGTGAGTGCTAC-3′ (R68Aforward), 5′-GTAGCACTCACGCATCTAGCAATACGTGTCCTTTCAATATC-3′ (R68reverse), 5′-TCCCAACCTAATGCTAATGTCAACATATCACAACGAAAGTGATGA-3′ (S124Tforward), 5′-CTTCATCACTTTCGTTGTGATATGTTGACATTAGCATTAGGTTGG-3′ (S124Treverse); where the underlined nucleotides generate the mutation. Supercompetent XL-1-Blue cells were transformed according to the manufacturer's instructions, and the plasmids were purified to confirm the sequence modifications by sequencing.

### Expression and purification of recombinant proteins

Subconfluent 293T cells in 35-mm diameter wells (Orange Scientific) were transfected with 2 mg plasmid DNA and 6.0 ml Fugene 6 (Roche Biochemicals) in Dulbecco's modified Eagle's medium (DMEM, Invitrogen) without FCS. The medium was harvested after 72 h. Pooled supernatants were cleared by centrifugation, concentrated 10-fold by filtration on 10000 NMWL membranes (Millipore), ultracentrifuged at 140,000 g before use, and finally stored at −80°C. Concentrated culture supernatants were analyzed by western blotting on a Hybond ECL membrane (GE healthcare) using an anti-V5 primary antibody (Invitrogen), an IgHRP conjugate as secondary antibody and the ECL detection reagent (GE healthcare) according to the manufacturer's instructions. The coding regions of Ir-LBP were amplified by PCR (94°C for 30 s, 56°C for 30 s, 72°C for 1 min.; 30 cycles) using ExTaq DNA Polymerase (Taqara). The PCR product was inserted into the pBlueBac4.5/V5-His Topo vector (Invitrogen) in frame with the coding sequence of the V5 and His epitopes at the C-terminus. Recombinant baculoviruses were generated by recombination between pBlueBac/*Ir-LBP* and Bac-N-Blue linear DNA virus (Invitrogen). Recombinant viruses were selected and amplified according to the manufacturer's instructions. Sf9 cells were infected with a high-titer stock of recombinant baculovirus and were incubated for 72 h at 27°C in Sf900 II serum-free medium (Invitrogen). Recombinant Ir-LBP proteins were purified from the cell culture supernatant by affinity chromatography on a His-Trap column (GE Healthcare). Proteins were recovered in 50 mM NaH_2_PO4 buffer (pH 7.5) containing 300 mM NaCl and 250 mM of imidazole.

### Binding assays

The dissociation constant was measured in PBS (pH 7.4) using purified Ir-LBP and an increasing amount of ^3^H-LTB4. Non-specific binding was determined by measuring bound radioactivity in the absence of Ir-LBP. Protein precipitation with polyethylene glycol 8000 was used to separate bound from free histamine [52]. Protein-bound radioactivity was determined with a Wallac 1409 scintillation counter. Saturation experiments were analyzed by non-linear regression using the curve fitting program GraphPad Prism® (GraphPad software, San Diego, CA, U.S.A.), B = (Bmax [F])/KD+[F]), where B is the amount of bound ligand at equilibrium, Bmax is the maximum number of binding sites, [F] is the concentration of free ligand and KD is the ligand dissociation constant. Competitive binding assays were performed using purified Ir-LBP with 1 nM of ^3^H-LTB4 and an increasing amount of unlabelled competitor for 2 h.

### Cell sorting, culture, and treatment for apoptosis assays

Human blood neutrophils were obtained from buffy coats (Transfusion Center, Liege, Belgium). Neutrophils were separated from mononuclear cells by density centrifugation (Histopaque; Sigma-Aldrich). Contaminating erythrocytes were removed from the neutrophil fraction by hypotonic lysis. Neutrophil purity, determined by counting cytospin preparations stained with Diff-Quick (Dade Behring), was always >95%. Neutrophils were cultured at a density of 2×10^6^ cells/ml in RPMI-1640 supplemented with 1% glutamine, 10% FCS, 50 µg/ml streptomycin, and 50 IU/ml penicillin (all from Gibco BRL). Neutrophils were cultured in the absence or presence of 10^−7^ M LTB4 (Sigma-Aldrich) and/or 0.2–2 µg/ml Ir-LBP.

### Apoptosis assays

Apoptosis was assessed by staining with Annexin-V-FITC and propidium iodide (PI) using the Annexin-V-FLUOS staining kit (Roche), following the manufacturer's recommendations. Flow cytometry analyses were performed with a FACSAria™ (BD Biosciences).

### Cells and culture for transendothelial assay

Ea.hy926 cells, an endothelial cell line derived from the human umbilical vein, were used. For the assay. These cells closely resemble HUVEC and maintain several characteristics of differentiated endothelium. The cells were allowed to reach confluence in chambers containing Dubelcco's Modified Eagle's Medium (DMEM) (Cambrex), supplemented with 10% Fetal Calf Serum, 2 mM L-glutamine, 100 U/ml penicillin, 100 µg/ml streptomycin and HAT (1000 µM hypoxanthine, 0.4 µM aminopterin, 16 µM thymidine).

#### Leukocyte-enriched fractions

Cells were prepared from peripheral blood from one healthy volunteer. Citrated blood was mixed with an equal volume of 6% dextran/0.9% NaCl solution and allowed to stand for 1 h at room temperature. Cell fractions were then recovered and spun at 1150 rpm for 12 min at 4°C. Pellets were re-suspended in ammonium chloride and, after 15 min, spun at 1300 rpm for 6 min at 4°C. This step was repeated until no red blood cells remained. Purity of preparations was >75% neutrophils as judged by Cell-dyn 1600 (Abbott) and by morphological examination with May-Grünwald Giemsa staining.

### Transendothelial assay

The protocol described by Nohgawa et al. was used for this test. In brief, isolated cells were re-suspended at a minimum concentration of 5×10^6^ cells/ml in RPMI containing 10% FBS and added to the upper chamber above the endothelial monolayer. We used the same medium with or without LTB4 (10E-7 M) and/or Ir-LBP (0.2 to 5 µg/ml). After 2 h at 37°C, the upper chambers were removed and cells in lower chambers were counted.

To be sure that the observed effect was really linked to neutrophils, and not to other concurrent cell types, we evaluated the cell type in the lower chamber by morphological examination with May-Grünwald Giemsa staining (data not shown). Each time, there were more than 99% neutrophils.

### siRNA silencing in 293T cells

Three siRNA (5′-AGUGCUACAUUGCGAUACATT-3′, 5′-AUGCUAAUGUCAUCAUAUCTT-3′ and 5′-GGUUAUAUUGGACCUUGUATT-3′; Sigma, Belgium) were designed to target specifically Ir-LBP mRNA. 293T cells were co-transfected with 500 ng of Ir-LBP/pCDNA3.1/V5-His-TOPO and 100 ng of each of the siRNA using the X-tremeGENE siRNA transfection reagent (Roche) according to the manufacturer's recommendations. Seventy-two h post transfection, culture supernatants were harvested, and the protein expression was analyzed by Western blot.

### *Ex vivo* SiRNA silencing in salivary gland extracts

The salivary glands from 10 partially (5-day) fed female ticks were incubated for 6 h at 37°C in the presence of 5 µg of control siRNA duplexes (Eurogentec) or a combination of the 3 Ir-LBP siRNA or buffer alone in a total volume of 200 µl of incubation buffer TC-199 (Sigma) containing 20 mM MOPS, pH 7.0.

### RT-PCR analysis to confirm gene silencing

Messenger RNA from salivary gland extracts was isolated by oligo-dT chromatography (MicroFastTrack 2.0 mRNA Isolation Kit, Invitrogen). Reverse transcription was routinely performed in a 20 µl standard RT reaction mixture according to the manufacturer's instructions (First-Strand cDNA Synthesis System, Invitrogen) using the oligo dT primer. PCR was routinely performed in 50 µl of standard Takara buffer containing 2.5 U of Taq polymerase (Takara Ex Taq, Takara, Japan), 10 pmoles of each primer, and 2.5 nmoles of each dNTP (Takara). PCR cycling conditions were as follows: 30 cycles of 95°C 30 s/58°C 30 s/72°C 1 min 30 s preceded by an initial 4 min denaturation step at 95°C and followed by a final 10 min extension at 72°C. Primers (sense-primer: 5′- GCCACCATGCTTAGAATAGCGGTGGTTGC-3′ and anti-sense primer: 5- CTCGGGTCCCTTGCGTTTGCA-3′) designed to amplify the Ir-LBP open reading frame were used to perform RT-PCR analysis of the transcripts. A pair of primers designed to amplify a 1,131 bp fragment from the actin open reading frame (sense-primer; 5′-ATGTGTGACGACGAGGTTGCC-3′ and anti-sense primer; 5′-TTAGAAGCACTTGCGGTGGATG-3′) were used as controls. 10 µl of the PCR reactions were analyzed on a 2% agarose gel.

### Tissue preparation, immunohistochemistry and morphometry

Mouse skin fragments with 5-day engorged ticks were embedded in TissueTek (Sakura) and stored at −20°C. Cryosections (10 µm-thick) were placed on poly-L-lysine coated slides and air-dried. Sections were then fixed in PBS containing 1% paraformaldehyde (PFA) for 10 min at room temperature and stored at −20°C.

Neutrophils were detected using the indirect biotin-streptavidin immunoperoxidase technique on skin sections containing tick rostra. Briefly, slides were rehydrated, washed 2 times for 2 min with H_2_O_2_ and incubated with a 1/100 dilution of anti-mouse neutrophil antibody (NIMP-R14; Abcam) for 1 h at room temperature. They were rinsed 3 times with PBS and incubated with biotin-conjugated anti-rat IgG for 1 h at room temperature. After 3 washes, they were incubated with streptavidin HRP-conjugate (Dako Cytomation) for 30 min at room temperature. HRP was revealed with AEC (Zymed). The sections were then counterstained with Carrazi haematoxylin and mounted in glycergel (Dako Cytomation).

Apoptotic cells were detected using ApopTag kit (Chemicon) according to the manufacturer's instructions.

We used a morphometrical technique to quantify labeled surfaces. Entire skin sections were digitalized. Labeled surfaces were measured using ImageJ software (National Institutes of Health), in 5 different zones per skin section. The ratios between the labeled surface and the total zone surface were calculated and the means of these ratios were compared between mouse skins infested with a tick treated with siIr-LBPRNA and mouse skins infested with control uninjected ticks or injected with PBS or siControlRNA. The values were compared by nonparametrical statistical analysis (Mann-Whitney test).

### Statistical analysis

Data are presented as means±standard deviation (SD). The differences between mean values were estimated using an ANOVA with subsequent Fisher's protected least significant difference tests. A value of *p* <0.05 was considered significant. The results presented are representative of three similar experiments.

## Supporting Information

Figure S1 Top view of Ir-LBP (center), LIR2 (right) and Ra-HBP2 (1QFT) (left). Negative and positive residues are in red and blue, respectively. The proteins are positioned as indicated by the ribbon representation of Ir-LBP.
(9.45 MB TIF)

Figure S2 Residues (represented as sticks and balls) of the cavity of LIR2 (left) and Ir-LBP (right) capable of interacting with a ligand. Proteins are represented by a ribbon, colored by secondary structure succession. Positive and negative residues are in dark blue and red, respectively, hydrophilic and hydrophobic residues are light blue and orange respectively, and Tyr is green.
(8.22 MB TIF)
