# Supplementary figures and images for: Ir-LBP, an Ixodes ricinus Tick Salivary LTB4-Binding Lipocalin, Interferes with Host Neutrophil Function
Source: PLoS One. 2008 Dec 19;3(12):e3987. doi: 10.1371/journal.pone.0003987 (PMC2600610; doi:10.1371/journal.pone.0003987)

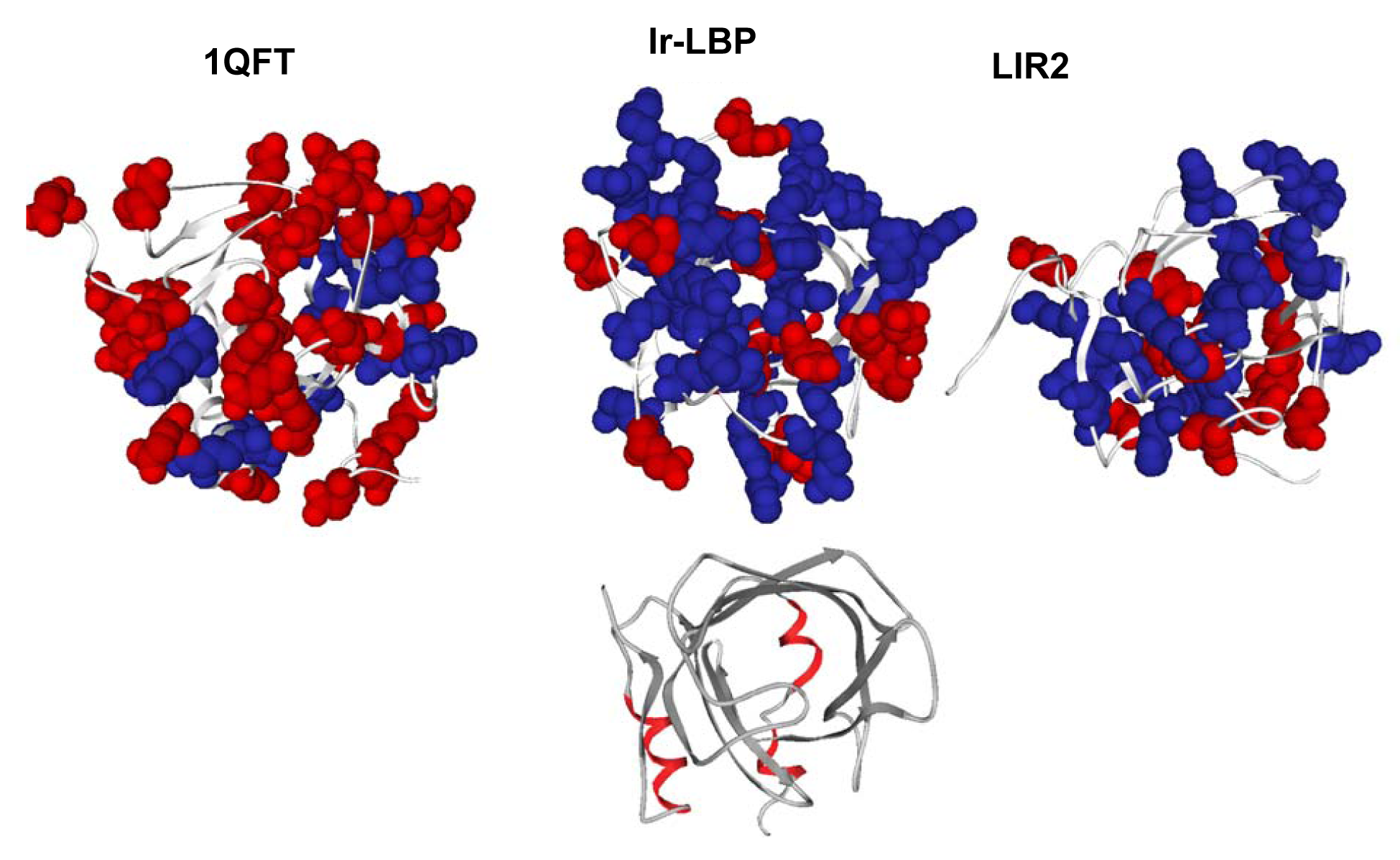

Supplement: Figure S1 — Top view of Ir-LBP (center), LIR2 (right) and Ra-HBP2 (1QFT) (left). Negative and positive residues are in red and blue, respectively. The proteins are positioned as indicated by the ribbon representation of Ir-LBP. (9.45 MB TIF) [file pone.0003987.s001.tif]

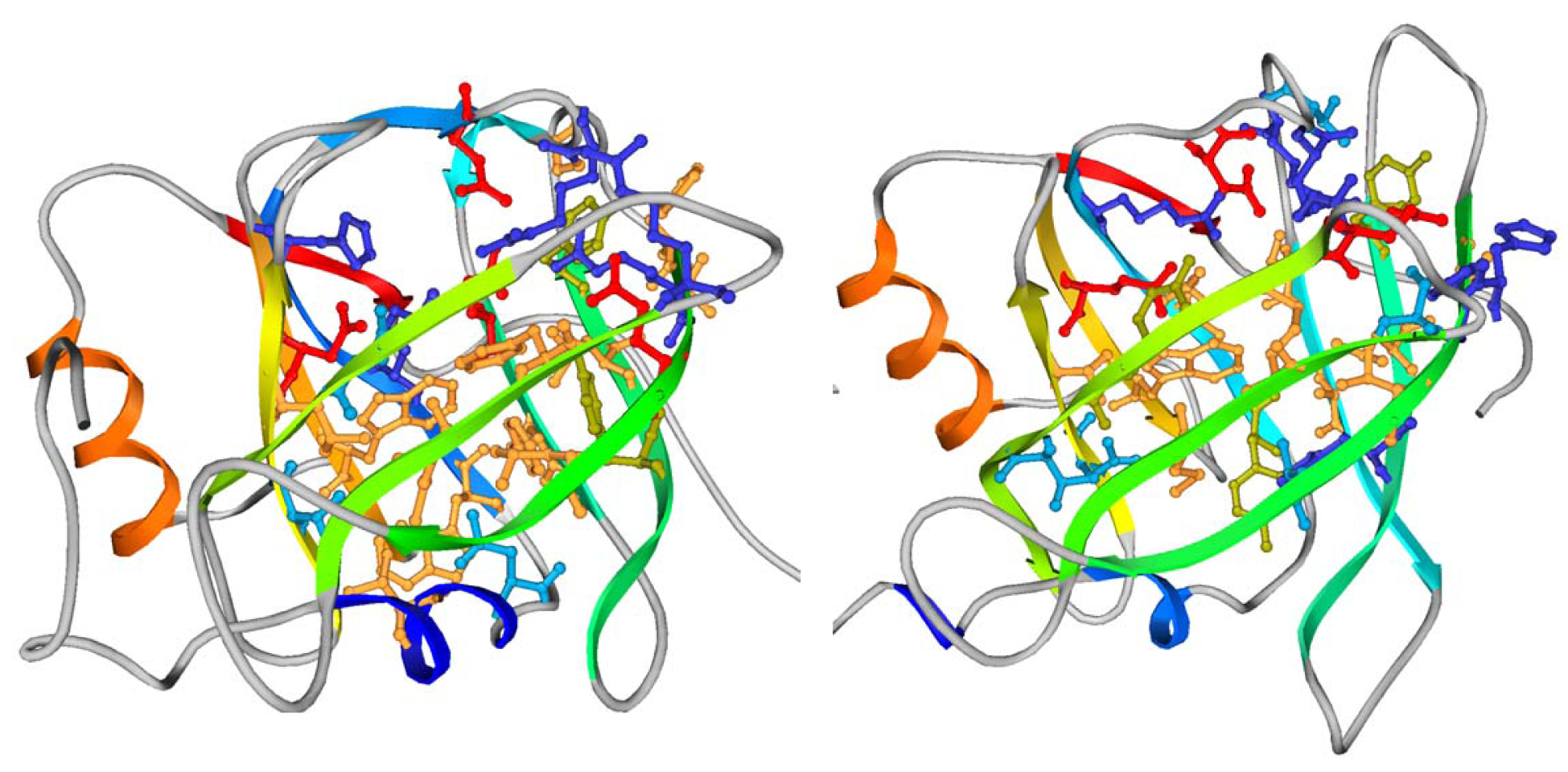

Supplement: Figure S2 — Residues (represented as sticks and balls) of the cavity of LIR2 (left) and Ir-LBP (right) capable of interacting with a ligand. Proteins are represented by a ribbon, colored by secondary structure succession. Positive and negative residues are in dark blue and red, respectively, hydrophilic and hydrophobic residues are light blue and orange respectively, and Tyr is green. (8.22 MB TIF) [file pone.0003987.s002.tif]
